# Acute and Long-Term Consequences of COVID-19 on Arterial Stiffness—A Narrative Review

**DOI:** 10.3390/life12060781

**Published:** 2022-05-25

**Authors:** Ioana Mădălina Zota, Cristian Stătescu, Radu Andy Sascău, Mihai Roca, Larisa Anghel, Alexandra Maștaleru, Maria Magdalena Leon-Constantin, Cristina Mihaela Ghiciuc, Sebastian Romica Cozma, Lucia Corina Dima-Cozma, Irina Mihaela Esanu, Florin Mitu

**Affiliations:** 1 Department of Medical Specialties I, Faculty of Medicine, Grigore T. Popa University of Medicine and Pharmacy, 700111 Iași, Romania; ioana-madalina.chiorescu@umfiasi.ro (I.M.Z.); cristian.statescu@umfiasi.ro (C.S.); radu.sascau@umfiasi.ro (R.A.S.); mihai.c.roca@umfiasi.ro (M.R.); larisa.anghel@umfiasi.ro (L.A.); alexandra.mastaleru@umfiasi.ro (A.M.); maria.leon@umfiasi.ro (M.M.L.-C.); cozma.dima@umfiasi.ro (L.C.D.-C.); mitu.florin@yahoo.com (F.M.); 2 Pharmacology, Clinical Pharmacology and Algeziology, Department of Morpho-Functional Sciences II, Faculty of Medicine, Grigore T. Popa University of Medicine and Pharmacy, 700111 Iași, Romania; 3 Department of Surgery (II), Faculty of Medicine, Grigore T. Popa University of Medicine and Pharmacy, 700111 Iași, Romania; sebastian.cozma@umfiasi.ro

**Keywords:** SARS-CoV-2, COVID-19, arterial stiffness, pulse wave velocity, cardiovascular risk, endotheliitis

## Abstract

The severe acute respiratory syndrome coronavirus 2 (SARS-CoV-2) is responsible for the ongoing global coronavirus (COVID-19) pandemic. Although initially viewed as an acute respiratory illness, COVID-19 is clearly a complex multisystemic disease with extensive cardiovascular involvement. Emerging evidence shows that the endothelium plays multiple roles in COVID-19 physiopathology, as both a target organ that can be directly infected by SARS-CoV-2 and a mediator in the subsequent inflammatory and thrombotic cascades. Arterial stiffness is an established marker of cardiovascular disease. The scope of this review is to summarize available data on the acute and long-term consequences of COVID-19 on vascular function. COVID-19 causes early vascular aging and arterial stiffness. Fast, noninvasive bedside assessment of arterial stiffness could optimize risk stratification in acute COVID-19, allowing for early escalation of treatment. Vascular physiology remains impaired at least 12 months after infection with SARS-CoV-2, even in otherwise healthy adults. This raises concerns regarding the extent of arterial remodeling in patients with preexisting vascular disease and the potential development of a persistent, chronic COVID-19 vasculopathy. Long-term follow up on larger cohorts is required to investigate the reversibility of COVID-19-induced vascular changes and their associated prognostic implications.

## 1. Introduction

The severe acute respiratory syndrome coronavirus 2 (SARS-CoV-2) is a single stranded, positive-sense, enveloped RNA virus, responsible for the current coronavirus disease 2019 (COVID-19) pandemic [1,2]. Although initially described as an acute respiratory disease, COVID-19 is now considered a complex multisystemic disease, with potential long-term consequences described in up to 25% of patients (long COVID-19, post-COVID-19 syndrome) [3].

**Figure 1:**
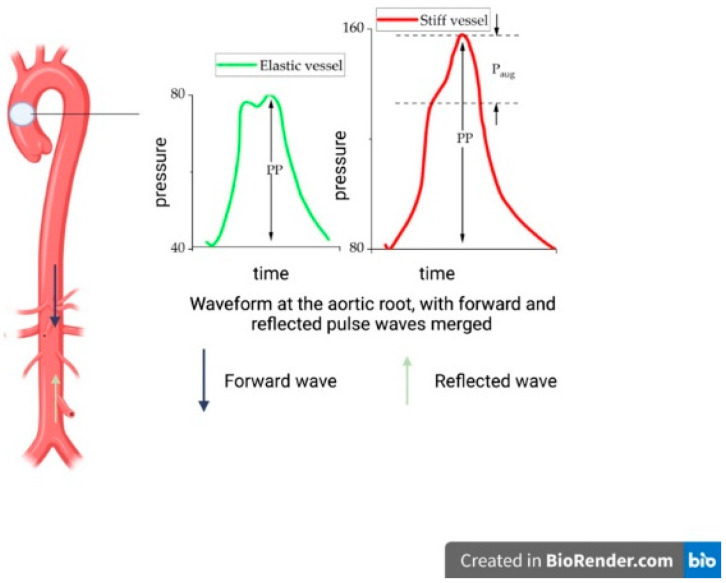
The concept of arterial stiffness. In stiff vessels, the reflection of the pulse wave occurs prematurely, during systole, leading to an early merger of the forward and reflected pulse waves, isolated systolic hypertension, adverse afterload pattern, reduced coronary perfusion and organ damage in low resistance vascular beds.

Arterial stiffening, although naturally associated with aging, can be accelerated by associated respiratory, metabolic and cardiovascular comorbidities (Figure 1) [4]. The chronic increase in afterload precipitates left ventricular remodeling and the development of heart failure [5]. As such, arterial stiffness and the modern concept of early vascular aging have been introduced as major determinants of vascular health. Arterial stiffness can be assessed in both muscular and elastic vessels, cross-sectionally or longitudinally, but in most cases, arterial stiffness is measured as the pulse wave travel velocity in a predefined segment of the aorta or any other large artery of the human body [6]. In addition, arterial stiffness can also be estimated using different formulae that employ arterial pressure and diameter [7]. Surrogate parameters for arterial stiffness assessment include pulse pressure (PP) [8], arterial compliance, distensibility or impedance, which can be used to compute arterial elastic modulus, a direct measure of parietal stiffness [6]. Systolic pressure augmentation, also known as the augmentation index, compares brachial artery pressure with central aortic blood pressure; although dependent on arterial stiffness, systolic pressure augmentation is also influenced by several other factors, especially heart rate [9]. Although arterial stiffness is a powerful prognostic marker, it is not routinely performed in clinical practice, partially due to the variety of methods and devices that claim to assess arterial stiffness and vascular age [10]. PWV is commonly calculated as the ratio between the distance and the pulse wave travel time for the pulse wave between the proximal and distal measurement sites. A direct, invasive aortic PWV measurement via pressure catheter recordings, offering a comprehensive anatomical delineation and correct estimation of pulse wave transit time, is rarely used in clinical practice due to cost and complexity. However, aside from the high cost and low availability, a high temporal resolution is required to accurately compute through-plane MRI signaling. Due to technical difficulties in assessing aortic PWV, its surrogate, carotid-femoral PWV (cfPWV) has become the gold standard for evaluation of arterial stiffness as recommended by current guidelines [6]. Other surrogates are brachial-ankle PWV (baPWV) and estimated PWV (ePWV) (equation derived from the Reference Values for Arterial Stiffness Collaboration [11,12]). The current guidelines for the management of arterial hypertension (HTN) [13] list a carotid-femoral PWV of >10 m/s as a feature of asymptomatic HTN-mediated organ damage, but with a weak class of recommendation.

Hypertension and age are major determinants of vascular stiffness and also independent risk factors for COVID-19 mortality [14]. Moreover, diabetes, obesity, smoking and dyslipidemia are known risk factors for endothelial dysfunction and for worse COVID-19 outcomes, and low-grade inflammatory components have regularly been labelled in the physiopathology of early vascular aging [15,16]. Therefore, a noninvasive arterial stiffness assessment could help optimize risk stratification in COVID-19 patients, which could favor a more aggressive treatment approach in “high-risk” patients. The scope of this review is to summarize available data on the acute and long-term consequences of COVID-19 on arterial stiffness and other parameters of vascular function.

## 2. Materials and Methods

The population targeted in the following review consists of data from the literature regarding patients of all ages with current or previous SARS-CoV-2 infection, isolated or compared to a control group of patients without a previous COVID-19 diagnosis. The primary intervention was an arterial stiffness assessment, either isolated or accompanied by the additional evaluation of endothelial and cardiac dysfunction.

### 2.1. Electronic Search Strategy

We conducted a comprehensive literature review of the articles currently available in the EMBASE, MEDLINE and PubMed databases, according to PRISMA guidelines. We used the following keywords: “COVID-19”, “SARS-CoV-2”, “arterial stiffness”, “PWV” and “pulse wave velocity”. This review was carried out according to the Preferred Reporting Items for Systematic Review and Meta-Analysis (PRISMA) checklist [17]. We applied the following selection criteria:

- Study type: retrospective, cross-sectional or prospective analysis, case reports and case series;
- Language: English;
- Types of participants: patients of all ages with current or previous SARS-CoV-2 infection;
- Follow-up duration: without restrictions.
- Outcome: COVID-19 severity and mortality.

Reviews, studies available only as abstracts (including conference abstracts) and dissertations were excluded from this analysis.

### 2.2. Arterial Stiffness Assessment

We selected studies evaluating arterial stiffness as well as other parameters of vascular function.

- Primary indicator: arterial stiffness assessment (pulse wave velocity: PWV; augmentation index: Aix; cardio-ankle vascular index: CAVI; arterial stiffness index: ASI; Young’s modulus of elasticity; pulse pressure: PP) [10,18,19,20];
- Secondary indicators: intima media thickness: IMT, endothelial dysfunction assessment (flow-mediated dilation: FMD; nitroglycerin mediated dilation: NMD; perfused boundary region) [21].

## 3. Results

We identified a total of 15 literature reports compatible with the beforementioned selection criteria: 4 cross-sectional studies, 3 retrospective studies, 6 prospective studies and 2 case reports.

### 3.1. Early Impact of COVID-19 on Vascular Stiffness

An increasing number of clinical studies have assessed the impact of COVID-19 on arterial stiffness (Table 1). As a first contribution to the subject, Ratchford et al. [22] observed that cfPWV (assessed by the ultrasound foot-to-foot electrocardiogram-gated method [18]) was 0.75 m/s higher in young adults with prior COVID-19 compared to healthy controls. An increase in cfPWV seemed gender-independent; however, small sample sizes limit the value of this conclusion. The authors additionally signal a discrepancy between impaired brachial FMD and a lack of change in brachial reactive hyperemia, which could suggest that microvascular dysfunction in COVID-19 is primarily mediated by diminished NO bioavailability [22].

The analysis of UK Biobank data, realized by Raisi-Estabragh et al. [23], on 70 COVID-19 patients and on 240 controls found the arterial stiffness index (ASI) (PulseTrace PCA2, CareFusion, San Diego, CA, USA) to be 0.6 m/s higher in COVID-19 fatalities (*n* = 8) compared to that of survivors (9.7 ± 2.7 m/s versus 9.1 ± 2.7 m/s, *p* < 0.05). However, the research did not find a significant association between ASI and COVID-19 status, mortality or critical care admission rates after age and sex adjustments [23]. In spite of these results, three other studies [14,24,25] have highlighted arterial stiffness as a short-term prognostic marker in COVID-19.

Schnaubelt et al. [25] compared PWV (BOSO ABI Systems 100 PWV, Bosch & Sohn GmbH, Jungingen, Germany) in 22 acutely ill COVD-19 patients and 22 acutely ill non-COVID-19 controls. BaPWV (brachial-ankle PWV) and cfPWV (carotido-femoral PWV) were independently associated with COVID-19 status in multiple regression models and were significantly higher in positive COVID-19 subjects compared to control groups (22 age- and sex-matched controls and 102 acutely ill COVID-19-negative controls). COVID-19 fatalities had greater baPWV and cfPWV (*p* = 0.004 and *p* = 0.05, respectively), and PWV was correlated with the length of hospital stay among the COVID-19 survivors, distinguishing arterial stiffness as an independent risk factor for clinical deterioration [25]. The results of two retrospective cohorts [14,24] additionally support this hypothesis. Rodilla et al. [14] analyzed 12,170 hospitalized COVID-19 patients and found that a pulse pressure ≥60 mmHg was an independent predictor for all-cause in-hospital mortality (adjusted OR 1.23, *p* = 0.0001). In another study, estimated PWV (ePWV) was higher among individuals hospitalized with COVID-19 compared to matched controls and offered prognostic information for 28-day mortality [24]. In a recent protocol, the prognostic value of ePWV was additional to current validated clinical predictors (4C Mortality score) [26].

**Table 1:** Overview of the available clinical studies assessing arterial stiffness in COVID-19.

	Study Type	Number of Subjects	Arterial Stiffness Assessment	Results
Ratchford et al. [22]	Cross-sectional case-control	11 young adults 3–5 weeks after a positive COVID-19 test 20 young healthy controls	cfPWV	cfPWV was higher in the SARS-CoV-2 group compared to controls (5.83 ± 0.62 m/s vs. 5.17 ± 0.66 m/s, *p* < 0.01);
			brachial FMD	FMD was lower in the SARS-CoV-2 group compared to controls (2.71 ± 1.21% vs. 8.81 ± 2.96%, *p* < 0.01);
Raisi-Estabragh et al. [23]	Retrospective case-control	70 COVID-19 patients (8 fatalities) 240 controls	ASI	No association between ASI and COVID-19 status, mortality or critical care admission rates in fully adjusted models; Higher ASI (lower arterial compliance) in COVID-19 fatalities
Rodilla et al. [14]	Retrospective observational cohort	12170 hospitalized COVID-19 patients	PP	PP ≥ 60 mmHg was an independent predictor for all-cause in-hospital mortality (OR 1.23, *p* = 0.0001);
Stamatelopoulos et al. [24]	Retrospective, longitudinal cohort	737 COVID-19 patients (184 deceased and 553 survivors) 934 controls	ePWV	ePWV progressively increased across the control group (9.97 m/s), COVID-19 survivors (11.0 m/s) and fatalities (13.9 m/s) (average increase/group 1.89 m/s, *p* < 0.001); ePWV provided additional prognostic value over the 4C Mortality score (optimal prognostic value 13 m/s);
Schnaubelt et al. [25]	Cross-sectional case-control	22 COVID-19 patients 22 controls	cfPWV	cfPWV was higher in COVID-19-patients vs. controls (14.3 m/s vs. 11.0 m/s, *p* = 0.007) cfPWV was higher among COVID-19 fatalities vs. survivors (*p* = 0.056); cfPWV was correlated with hospital stay duration in COVID-19 survivors (r = 0.689, *p* = 0.019);
			baPWV	maximum baPWV was higher among COVID-19 fatalities vs. survivors (*p* = 0.004);
Szeghy et al. [27]	Cross-sectional case-control	15 young adults 3–4 weeks after a positive COVID-19 test 15 young healthy controls	Aortic Aix	Aortic Aix higher in the SARS-CoV-2 group compared to controls (13 ± 9% vs. 3 ± 13%) *p* < 0.05;
			Carotid stiffness	Carotid stiffness was lower in SARS-CoV-2 group (2.6 ± 1 m/s) compared to controls (5 ± 1 m/s), *p* < 0.05;
			Young’s modulus of elasticity	Young’s modulus was higher in SARS-CoV-2 (576 ± 224 kPa) vs. controls (396 ± 120 kPa), *p* < 0.05;
			cIMT	cIMT was similar between groups (0.42 ± 0.06 vs. 0.44 ± 0.08 mm; *p* > 0.05);
Aydin et al. [28]	Prospective case-control	65 COVID-19 patients (25 moderate–severe cases, 40 mild cases) 50 healthy controls	R-CAVI	R-CAVI higher in COVID-19 vs. controls (9.6 ± 2.4 vs. 8.5 ± 1.1, *p* = 0.004) and moderate–severe vs. mild cases (10.8 ± 3.4 vs. 8.8 ± 0.9, *p* = 0.008); R-CAVI (cut off-point > 8.75) distinguished between mild and moderate–severe COVID-19 (76% sensitivity and 56% specificity);
			L-CAVI	L-CAVI higher in COVID-19 vs. controls (9.4 ± 2.7 vs. 8.5 ± 1.2, *p* = 0.01) and moderate–severe vs. mild cases (10.7 ± 3.6 vs. 8.5 ± 1.5, *p* < 0.001); L-CAVI (cut-off point > 8.5) distinguished between mild and moderate–severe COVID-19 (88% sensitivity and 58% specificity);
Nandadeva et al. [29]	Prospective case-control	16 young adults at least 4 weeks after COVID-19 diagnosis (8 with persistent symptoms) 12 healthy controls	cfPWV Aix Brachial artery FMD and reactive hyperemia	cfPWV, Aix, FMD and peak blood velocity following cuff release were similar between COVID-19 patients and controls;
			FMD	FMD (but not cfPWV) was lower in patients with persistent symptoms (3.8 ± 0.6%) compared to asymptomatic patients (6.8 ± 0.9%; *p* = 0.007) and controls (6.8 ± 0.6%; *p* = 0.003);
Jud et al. [30]	Cross-sectional case-control	14 post COVID-19 patients 14 controls with atherosclerotic cardiovascular disease 14 healthy controls	PWV	PWV was higher in COVID-19 patients than in healthy controls (10.75 m/s versus 5.70 m/s, *p* < 0.001)
			Aix	Aix was higher in COVID-19 patients compared to healthy controls (22% versus 4%, *p* = 0.009);
			carotid, axillary and superficial femoral artery IMT	Common carotid, axillary and superficial femoral IMT were higher in COVID-19 patients (0.59 mm, 0.58 mm and 0.54 mm, respectively) compared to healthy controls (0.44 mm, 0.40 mm and 0.40 mm, respectively), *p* < 0.001; PWV, Aix and IMT were similar in COVID-19 patients and patients with atherosclerotic cardiovascular disease, except for axillary artery IMT, which was lower in the COVID-19 group (*p* = 0.01);
			FMD, NMD	FMD and NMD were similar within all three groups;
Kumar et al. [31]	Prospective observational	23 mild, 21 moderate and 20 severe COVID-19 cases without comorbidities	cfPWV	cfPWV was significantly lower in mild COVID-19 cases compared to moderate and severe cases, respectively (829.1 ± 139.2 cm/s versus 1067 ± 152.5 cm/s, *p* < 0.0001 and 1416 ± 253.9 cm/s, *p* < 0.0001);
Ciftel et al. [5]	Prospective case-control	38 cases of post COVID-19 MIS-C 38 controls	Aortic strain Aortic distensibility	Children with MIS-C had lower aortic distensibility (8.90 ± 4.3 vs. 13.91 ± 3.7; *p* < 0.01) and aortic strain (11.64 ± 5.3 vs. 17.07 ± 4.4; *p* < 0.01) vs. controls;
			Brachial FMD	Brachial FMD was correlated with arterial stiffness and left ventricular systolic function;
Lambadiari et al. [32]	Prospective, observational case-control	24 mild, 23 moderate and 23 severe COVID-19 cases, 4 months after diagnosis 70 hypertensive controls 70 healthy controls	cfPWV	PWV was higher in COVID-19 patients and in hypertensive controls compared to healthy controls (12.09 ± 2.50; 11.92 ± 2.94; vs. 10.04 ± 1.80m/s, *p* = 0.036 and *p* = 0.045, respectively);
			FMD	FMD was similar in COVID-19 patients, and hypertensive controls had similar FMD (5.86 ± 2.82% vs. 5.80 ± 2.07%, *p* = 0.872), while both groups had lower FMD values than healthy controls (9.06 ± 2.11%, *p* = 0.002 and *p* = 0.002, respectively;
Ikonomidis et al. [33] *	Prospective, observational case-control	24 mild, 23 moderate and 23 severe COVID-19 cases, 12 months after diagnosis 70 hypertensive controls 70 healthy controls	cfPWV	cfPWV remained persistently higher in COVID-19 patients versus controls 12 months after infection (11.19 ± 2.53 m/s versus 10.04 ± 1.80 m/s, *p* = 0.05);
			FMD	FMD values remained persistently higher in COVID-19 patients versus controls 12 months after infection (6.49 ± 2.25% versus 9.06 ± 2.11%, *p* < 0.001);
			central SBP	Central SBP remained persistently higher in COVID-19 patients versus controls 12 months after infection (127.56 ± 15.26 mmHg versus 117.89 ± 18.85 mmHg, *p* = 0.003);

COVID-19: coronavirus disease 2019; cfPWV: carotid-femoral pulse wave velocity; SARS-CoV-2: severe acute respiratory syndrome coronavirus 2; PP: pulse pressure; ASI: arterial stiffness index; baPWV: brachial-ankle pulse wave velocity; Aix: augmentation index; IMT: intima media thickness; cIMT: carotid intima-media thickness; MIS-C: multisystem inflammatory syndrome in children; FMD: flow mediated dilation; CAVI: cardio-ankle vascular index; R-CAVI: right-cardio-ankle vascular index; L-CAVI: left-cardio-ankle vascular index; ePWV: estimated pulse wave velocity; NMD: nitroglycerin mediated dilation; SBP: systolic blood pressure; *: 12 months follow-up of Lambadiari et al. [32].

Szeghy et al. [27] reported that the carotid stiffness and aortic augmentation index (SphygmoCor—AtCor Medical, Sydney, Australia) are higher among young adults with recent COVID-19 (*n* = 15) than among healthy controls (*n* = 15). COVID-19 patients also had greater Young’s modulus of elasticity, indicating an increased risk of developing arterial hypertension over the following 3 years. Despite previous reports of acute increases in carotid intima thickness during hyperinflammatory states, cIMT had similar values in cases and controls, possibly due to the mild COVID-19 clinical presentation among the analyzed patients. Indeed, a prospective case-control analysis showed that the left and right CAVI (VaSera VS-1000-Fukuda-Denshi Company Ltd., Tokyo, Japan) were significantly higher in moderate–severe COVID-19 versus mild COVID-19 [28]. The left and right CAVI were more impaired among COVID-19 patients, and cut-off values of >8.5 and >8.75, respectively, could predict disease severity [28].

Vascular function can be influenced not only by COVID-19 severity in the acute phase, but also by the persistence of symptoms. Supporting this hypothesis, Nandadeva et al. [29] showed that peripheral micro- and macrovascular function (reactive hyperemia and brachial FMD) were impaired only among young adults with lingering COVID-19 symptoms (4 weeks after initial diagnosis). In contrast, cerebral vasomotor reactivity and central arterial stiffness (PWV, SphygmoCor, Atcor Medical, Sydney, Australia) were similar in all analyzed subgroups (symptomatic: *n* = 8, asymptomatic: *n* = 8 and controls: *n* = 12).

Available evidence shows that COVID-19 causes significant short-term alterations to vascular physiology even in otherwise healthy young adults. Judd et al. [30] reported substantial differences regarding PWV (Mobil-O-Graph, I.E.M., Aachen, Germany) and Aix, as well as carotid, axillary and superficial femoral IMT in patients 6 months after SARS-CoV-2 infection versus controls. Although vascular reactivity (FMD and NMD) did not significantly vary among the three analyzed subgroups, the authors additionally document persistent capillary changes (higher rates of capillary ramifications, capillary loss, bushy capillaries and capillary elongations) and disturbed arginine, kynurenine and homocysteine metabolism only among post-COVID-19 patients [30]. The prospective nonrandomized observational COSEVAST study [31] enrolled 64 patients without known comorbidities requiring hospitalization for COVID-19. Aix and cfPWV estimated from the brachial-ankle PWV (Periscope, Genesis Medical Systems, Hyderabad, India) gradually increased with COVID-19 severity (mild, moderate and severe, according to the National Institute of Health’s criteria), even after adjustments for potential confounding factors (weight, gender, mean arterial pressure and heart rate). The authors noted that the vascular damage in severe COVID-19 cases was comparable to that observed in long standing chronic diseases (coronary atherosclerosis, diabetes and renal failure) [31].

Although COVID-19 infection in children is usually mild, some patients develop MIS-C (multisystem inflammatory syndrome in children), a late complication hallmarked by hyperinflammation, fever and multiorgan dysfunction. Ciftel et al. [5] found that two echocardiography derived arterial stiffness markers (aortic strain and distensibility) were lower in MIS-C versus controls (*p* < 0.01). Interestingly, brachial FMD was correlated with markers of arterial stiffness and left ventricular systolic function [5].

### 3.2. Long Term Impact of COVID-19 on Vascular Stiffness

COVID-19 patients present persistent arterial stiffness and endothelial dysfunction at least 4 months after initial infection, as shown by Lambadiari et al. [32]. This interesting study showed that both cfPWV (Complior—Alam Medical, Vincennes, France) and brachial FMD were more impaired among patients with associated HTN and among patients with previous COVID-19 compared to healthy controls. These results suggest a long-term impact of COVID-19 on both arterial stiffness (vascular) and endothelial function. Coronary flow reserve (CFR), an early marker of endothelial dysfunction with prognostic implication, was lower among patients with associated HTN and COVID-19 patients compared to controls (*p* = 0.01 and *p* = 0.03, respectively). Moreover, the perfused boundary region of sublingual arterial microvessels with a diameter of 5–25 µm (PBR5-25), a marker of endothelial glycocalyx impairment, was higher in both COVID-19 and hypertensives compared to controls (*p* = 0.001 and *p* = 0.001). COVID-19 and hypertension seem to inflict a similar degree of vascular damage. The same study reported a significant association between persistent cardiovascular symptoms and poorer cfPWV, FMD, right and left ventricular strain values and MDA (oxidative stress). However, cfPWV did not vary with COVID-19 severity, suggesting that vascular dysfunction persists independently of initial disease severity, although this hypothesis should be confirmed in larger cohorts. At the 12-month follow-up [33], COVID-19 patients presented persistent arterial stiffness and endothelial dysfunction: cfPWV and central SBP remained significantly higher in COVID-19 patients compared to controls (*p* = 0.057 and *p* = 0.003, respectively), and PBR5-25 increased compared to the initial evaluation at 4 months. The authors reported significant improvements in oxidative stress (MDA levels), CFR and myocardial work parameters (myocardial wasted work and efficiency), as well as a borderline improvement in left ventricular strain, which, however, remained impaired compared to the controls [33]. Right heart function (right ventricular strain, tricuspid annular plane excursion) completely recovered at 12 months, possibly due to resolution of pulmonary lesions [33].

The ongoing CARTESIAN [34] study is a large longitudinal multicenter project that analyzes cfPWV, central hemodynamics as well as biomarkers of accelerated vascular aging, 6 and 12 months after confirmed SARS-CoV-2 infection. A pre-planned study extension aims to evaluate 10-year mortality causes, hospitalization rates and overall health status in COVID-19-positive patients.

### 3.3. Case Reports

Two case reports offer interesting insights regarding COVID-19-induced vasculopathy [35,36]. The Biostrap biosensor (Biostrap USA LLC, Duarte, CA, USA), a wrist worn device that records heart rate, respiratory rate, peripheral oxygen saturation and arterial stiffness via photoplethysmography, documented marked oscillations in arterial stiffness during SARS-CoV-2 infection in two patients from the first case report. The analysis showed a pattern of sharp decreases in arterial stiffness, inversely correlated with rises in pulse and respiratory rates [35]. Although the Biostrap biosensor was validated in 2018 for measurement of basic clinical variables [37], its accuracy in assessing arterial properties is unclear. The second case report documented a 0.3 m/s decrease in PWV (Mobil-O-Graph, I.E.M., Cockerill str., Stolberg, Germany) 6 weeks after COVID-19 in an otherwise healthy 24-year-old female [36]. Although both PWV values fell within the normal range, these results suggest that COVID-19 could impact endothelial and vascular function at a very early stage, even in asymptomatic patients [36].

## 4. Discussion

The concept of arterial stiffness reflects both the mechanical and functional properties of the arterial wall, reflecting the changes in blood pressure, flow and vascular diameter that occur with every heartbeat. The mechanical structure of the tunica media, namely the interaction between elastin (engaged at low blood pressure and distension) and inelastic collagen fibers (engaged at higher pressure and distension), is the main determining factor of arterial stiffness in large conduit vessels. Aging and vascular disease tend to reduce the number of functional elastic fibers and increase the inelastic collagenous component, which explains the natural increase observed with age in arterial stiffness (vascular senescence). Although the loss of elastic fibers, replacement fibrosis, collagen and elastin cross-linking and medial calcifications are the major determinants of vascular stiffness, arterial stiffness is also influenced by endothelial (dys)function (inflammation, oxidative stress and extracellular matrix turnover and modulation of smooth muscle tone in muscular arteries), reflecting a subtle intima-media interaction. As such, arterial stiffness variations can be classified as passive (due to elastin–collagen ratio and heart rate variability) and active (induced by nitric oxide, endothelin and vascular smooth muscle tone) [6,10].

Carotid-femoral PWV (cfPWV), the surrogate of PWV, has become the gold standard for arterial stiffness evaluation, as recommended by current guidelines, due to technical difficulties in assessing aortic PWV [6]. Commercially available systems (Complior [38], Sphygmocor [39], Pulsepen [40]) allow for the measurement of pulse wave transit time by simultaneous or ECG-gated recordings of carotid and femoral pulse wave signals. However, carotid-femoral pulse travel distance is estimated as 0.8× the distance between the proximal and distal measurement sites, generating a risk of bias and ambiguity. Although cfPWV is an evidence based cardiovascular risk predictor in both Europe and the United States [41,42], it excludes the ascending aorta and a segment of the aortic arch, which are vital elements for aortic buffering function. Furthermore, the cfPWV assessment is time consuming and difficult to implement in routine clinical practice. This has encouraged the development of other devices that record peripheral pulse wave signals via brachial or ankle pressure cuffs or finger plethysmography [43]. Brachial-ankle PWV (baPWV), finger-toe PWV and the cardiac-ankle vascular stiffness index are established/evidence-based cardiovascular risk predictors in Asian populations, but these have yet to demonstrate their prognostic value in United States’ and European cohorts [6]. However, such techniques analyze pulse wave travel velocities through both elastic and peripheral muscular arteries, which significantly reduces specificity, resulting in modest correlations with invasive arterial stiffness measurements [44]. Although the technique is largely disputed by experts in the field, some devices (Arteriograph [45], Mobil-O-Graph [46]) offer an indirect estimation of aortic PWV from waveform analysis from a single brachial pressure cuff recording [10]. Apart from novel ultrasound techniques (pulse wave imaging and shear wave elastography) that offer a local vascular stiffness assessment, a pulse pressure (defined as the difference between systolic and diastolic blood pressure values) value of ≥60 mmHg is a readily available surrogate marker for an arterial stiffness evaluation, especially in elderly individuals [8]. Computer tomography (CT) and fluorodeoxyglucose (FDG)-positron emission tomography (FDG-PET) have also been previously used for estimation of both arterial stiffness and cardiovascular risk. Although FDG-PET provides valuable insight regarding subclinical arterial inflammation and is associated with PWV [47,48], it has several limitations: limited availability, high costs and a somewhat high radiation exposure. Meanwhile, CT is broadly available, rather cheap and frequently used in the assessment of COVID-19 patients. CT perfusion imaging has recorded significant technical improvement [49,50], and several new indications in vascular imaging are in clinical investigation. The CT aortic stiffness index (CTASI) proved to be a robust measure of arterial stiffness [51]. CT is able to assess both the functional and anatomical components of vascular stiffness, including the coronary and descending aorta calcium scores. Dual source CT offers good spatial and temporal resolution, making ECG-gated CT imaging a promising area for further clinical studies of arterial stiffness assessment.

The classic concept of arterial stiffening refers to the impaired ability of large, elastic arteries to buffer the cyclical systolic and diastolic blood pressure variations [52]. More recently, the concept of peripheral artery stiffness has emerged. In comparison to central arteries, peripheral (muscular) arteries are inherently stiffer, causing a physiological gradient of stiffness across the vasculature observed in healthy individuals. Age-related arterial stiffening is much more prominent in large, elastic arteries compared to peripheral vessels, leading to a reversal in the physiological gradient of arterial stiffness in elderly patients [52]. Studies have yielded limited and conflicting results regarding the prognostic value of peripheral arterial stiffness [53,54,55,56,57]. However, the aortic to brachial stiffness ratio (aortic-brachial PWV ratio) could be a valuable predictor of the cardiovascular outcome [58,59,60].

### 4.1. (Cardio)vascular Involvement in COVID-19

**Figure 2:**
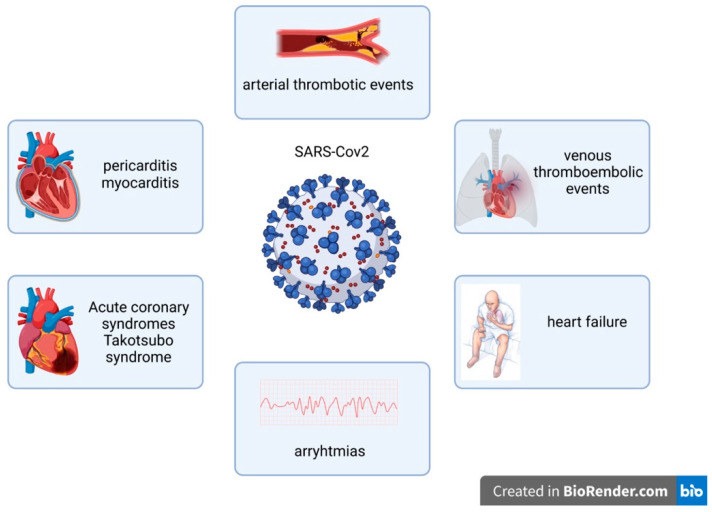
Cardiovascular complications of SARS-CoV-2.

COVID-19 causes a plethora of cardiovascular manifestations [61], ranging from arrythmias, asymptomatic myocardial injury, overt congestive heart failure and thrombo-embolic events (Figure 2) [62], attributable to the virus’ direct cytotoxic effect or to the systemic inflammatory cytokine storm [2]. Emerging evidence suggests that the endothelium is a primary target for SARS-CoV-2 [63,64]. Vascular endothelial cells express ACE2-R (angiotensin-converting enzyme 2 cellular receptor) and TMPRSS2 (transmembrane serine protease 2), which synergistically mediate SARS-CoV-2 entry in host cells (essential for SARS-CoV-2 pathogenicity) [65,66]. The infected endothelial cells demonstrate an increased production of proinflammatory cytokines and prothrombotic factors. The multiorgan failure observed in some COVID-19 cases is partly caused by vasculitis in multiple vascular areas [63,67]. Although two recently published papers argued that human endothelial cells do not express ACE2-R, Ma et al. demonstrated an ACE2-R-independent direct inflammatory activation of the endothelium, stating that other surface receptors (neuropilin-1, scavenger receptor B type 1 and CD147) could assist the direct cellular entry of SARS-CoV-2 [68].

Vascular endothelium integrity is critical for maintaining homeostasis and finetuning inflammatory and prothrombotic pathways. Direct invasion of and damage to vascular endothelium (endotheliitis) [63] cause apoptosis of endothelial cells with a subsequent loss of integrity and endothelial dysfunction (ED). Increased endothelial permeability leads to the exposure of subendothelial compounds, thrombin activation, fibrin production and capture of platelets, as well as an increased expression of cytokines and adhesion molecules for circulating inflammatory cells [69]. Dysfunctional endothelial cells reduce their prostacyclin synthesis (affecting the fine balance between vasodilation and vasoconstriction [70]) and overexpress adhesion molecules, which enhance platelet recruitment and activation. Thrombocyte-dependent recruitment of white blood cells leads to the formation of tricellular aggregates (endothelial cell-platelet-leucocyte), which impairs microvascular perfusion. Intravascular thrombosis damages endothelial cells, which aggravates the endothelial dysfunction and encloses a vicious feedback loop [2].

Direct invasion of and damage to endothelial cells (endotheliitis) [63], and subsequent endothelial dysfunction, are associated with poor outcome [64]. As such, the endothelium plays a dual role in COVID-19 physiopathology—a target organ for the infection and a mediator in the subsequent inflammatory and thrombotic cascades [64]. Endothelial dysfunction can extend past the acute phase, with reports of persistent oxidative stress and ED markers 4 months after COVID-19 diagnosis [32].

From a macrovascular perspective, evidence suggests that COVID-19 causes early vascular aging [5]. COVID-19-induced mitochondrial dysfunction, increased local production of reactive oxygen species (ROS) and subsequent oxidative telomere shortening have been proposed as other potential causes of cellular senescence and vascular stiffening [71]. Imbalance of the host redox status favors ED and chronic subintimal inflammation, which causes accelerated fragmentation of parietal elastin fibers and their replacement with rigid, fibrotic tissue [63,71]. As COVID-19-induced pulmonary fibrosis is only partially reversible [72], it has been postulated that arterial stiffening could be a long-term cardiovascular sequela for most patients, irrespective of COVID-19 severity [63].

The European Society of Cardiology endorses that close follow-up and further research is needed to address the potential therapeutic and prognostic implications of COVID-19-induced endotheliitis, recommending an arterial stiffness assessment as a marker of COVID-19 outcome and treatment monitoring [2]. Saeed et al. have recently argued that COVID-19 and arterial stiffness have a bidirectional cause–effect association [73]. COVID-19-induced vascular remodeling is favored by dysregulation of the neuro-hormonal systems, endothelial dysfunction, renal damage, altered lipid and glucose metabolisms and decompensated hypertension [73]. Pre-existing atherosclerosis is an independent risk factor for COVID-19 severity, and statins have been postulated to improve COVID-19 outcomes though their pleiotropic anti-inflammatory properties and a potential inhibition of SARS-CoV-2 proteases [74,75]. However, other studies failed to prove a substantial benefit with statin use [76,77], raising some safety concerns, especially since statin therapy upregulates ACE2 receptor expression [78], which could enhance SARS-CoV-2 entry into respiratory epithelial cells [66].

### 4.2. Consequences of COVID-19 on Arterial Stiffness

The available evidence shows that COVID-19 causes significant alterations to vascular physiology, even in otherwise healthy young adults. Cardiovascular involvement is central in COVID-19, and preexisting cardiovascular disease is associated with worse clinical outcomes. Systemic hyperinflammation reduces NO (nitric oxide) bioavailability, which increases vascular stiffness even in otherwise healthy individuals [79]. The endothelium is essential in vascular tone regulation and vascular remodeling, as well as in platelet aggregation and inflammation [5]. The two major causes of endothelial dysfunction comprise direct mechanical injury and inflammation (including autoantibodies and bacterial infection) [80], which can simultaneously occur in COVID-19. The importance of endothelial dysfunction in COVID-19 pathogenesis is supported by the fact that diabetes, obesity, smoking and dyslipidemia are known risk factors for ED and for worse COVID-19 outcomes [15,16]. As COVID-19 is associated with ED, altered lipid and glucose metabolism, as well as decompensated HTN, it induces early, accelerated atherosclerosis [73]. Furthermore, aging and a proinflammatory phenotype induced via angiotensin II signaling underlie the vicious circle of hypertension and arterial stiffening, which increases left ventricular afterload and impairs coronary perfusion [81,82].

The available reports show that some arterial stiffness parameters are correlated with length of COVID-19 hospital stay and are independent predictors for in-hospital and short-term COVID-19 mortality [14]. Lambadiari et al. [32] and their 12-month follow-up study [33] show that COVID-19 patients present persistent arterial stiffness and endothelial dysfunction.

Due to the limited number of patients enrolled in current, published reports, the gender-specific cardiovascular effects of SARS-CoV-2, as well as the impact of COVID-19 severity on arterial stiffness, need further studies. Associated comorbidities, especially chronic kidney disease, coronary and peripheral artery disease, cause significant, accelerated vascular remodeling and a prothrombotic state, increasing the risk of a COVID-19-negative outcome [83,84]. In our opinion, the prognostic significance of SARS-CoV-2-associated arterial stiffening in patients with preexisting alterations of vascular function should be analyzed in future dedicated cohorts. Vascular function impairment could depend not only on the severity of COVID-19 in its acute phase, but also on the persistence of symptoms. As such, clinicians should consider at least a basic screening of arterial stiffness in patients suffering from “long COVID-19”.

After the emergence of new SARS-CoV-2 strains, clinicians have noted a shift in COVID-19’s clinical presentation, with Omicron generating more upper respiratory tract symptoms and, apparently, fewer thrombotic complications [85]. In this context, the impact of COVID-19 on arterial stiffness could significantly vary according to the causal SARS-CoV-2 variant. This poses further difficulties in interpreting the current literature findings. The ongoing CARTESIAN study should clarify these aspects considering the impressive number of enrolled patients.

Nevertheless, COVID-19 seems to have a prolonged impact on cardiovascular function, and PWV appears useful in providing short- and long-term prognostic information regarding COVID-19 adverse clinical outcomes. A more prominent rise in PWV seems to be associated with unfavorable COVID-19 outcomes. Therefore, a noninvasive arterial stiffness assessment could help identify individuals at risk of clinical deterioration. Recent technological advances allow fast, noninvasive bedside assessment of arterial stiffness, which can be easily implemented in clinical practice, even in critically ill patients. Optimizing risk stratification using easily implemented arterial stiffness surrogates (ePWV, PP) could favor a more aggressive treatment approach and the application of novel therapies in “high-risk” patients.

Whether COVID-19-induced vasculopathy resolves after a couple of months or tends to evolve into a chronic vasculopathy with severe implications on cardiovascular morbimortality, requires long-term follow-up on large cohorts of patients, especially since “long COVID-19” is reported in up to 25% of cases [86].

## 5. Conclusions

COVID-19 causes early vascular aging and arterial stiffness. Future research should focus on screening, prevention and treatment of COVID-19 vasculopathy. A better understanding of COVID-19’s vascular involvement and its prognostic significance will help characterize COVID-19 in its entirety, which is an essential step in its successful management. Further studies are needed in order to investigate the reversibility of COVID-19-induced vascular changes and their impact on long-term prognosis, while keeping in mind that new, emerging variants could have completely different effects on vascular physiology.
